# Isolation, Characterization and Reverse Genetic System Establishment of a Highly Virulent PEDV Strain

**DOI:** 10.3390/v18080864

**Published:** 2026-08-07

**Authors:** Fan Zhang, Helu Liu, Linlong Ji, Yanyang Zhou, Heng Chen, Jiyong Zhou, Jinyan Gu

**Affiliations:** 1 MOA Key Laboratory of Animal Virology, Zhejiang Provincial Engineering Research Center of Animal Biological Products, Center for Veterinary Sciences, Zhejiang University, Hangzhou 310058, China; fanzhang20212021@163.com (F.Z.); 3220105200@zju.edu.cn (H.L.); jilinlong@outlook.com (L.J.); zyy19960604@163.com (Y.Z.);; 2 State Key Laboratory for Diagnosis and Treatment of Severe Infectious Diseases, First Affiliated Hospital, Zhejiang University, Hangzhou 310058, China

**Keywords:** porcine epidemic diarrhea virus, G2c genotype, reverse genetics

## Abstract

Porcine epidemic diarrhea virus (PEDV) G2c variants have recently emerged, posing significant challenges to swine health management. As a major coronavirus affecting the swine industry, PEDV exhibits extensive genetic variability, which has greatly complicated disease control. Current vaccines provide suboptimal protection under field conditions. Therefore, the isolation of recently circulating strains and the establishment of a robust reverse genetics system are critical for advancing the study of emerging variants and facilitating rational vaccine development. In this study, a PEDV field strain designated PEDV-BJ-2023 was isolated from diarrheic piglets in Guizhou, China. Phylogenetic analysis based on the complete genome and spike gene classified PEDV-BJ-2023 within the emerging G2c lineage. To facilitate functional studies, a full-length infectious cDNA clone was constructed using transformation-associated recombination cloning in yeast. Furthermore, an enhanced green fluorescent protein reporter virus was generated via CRISPR/Cas9-assisted homologous recombination by inserting an EGFP-2A cassette upstream of the nucleocapsid gene. The recombinant viruses displayed virion morphology and plaque characteristics similar to those of the parental wild-type PEDV-BJ-2023 strain, although the parental virus exhibited faster replication during the early stage of infection in vitro. In 5-day-old piglets, all three viruses caused severe diarrhea, weight loss, and intestinal lesions; however, recombinant viruses exhibited slightly reduced viral shedding and pathogenicity, with rPEDV-EGFP being the most attenuated. Notably, rPEDV-EGFP maintained stable EGFP expression over eight serial passages. This study establishes a reverse genetics platform for an emerging G2c PEDV strain and provides a stable fluorescent reporter virus, offering valuable tools for visualizing viral infection and investigating virus–host interactions.

## 1. Introduction

Porcine epidemic diarrhea virus (PEDV), a member of the genus *Alphacoronavirus* within the family *Coronaviridae* [1], is one of the most economically important viral pathogens in the swine industry [2], particularly in China [3,4]. PEDV infection causes severe acute enteric disease characterized by diarrhea, vomiting, and dehydration [5], leading to high mortality in neonatal piglets [6] and substantial economic losses worldwide. Since its first identification in the United Kingdom in 1977 [7], PEDV has spread globally and become endemic in China after its introduction in the 1980s [8,9,10]. A large-scale outbreak caused by highly virulent PEDV occurred in southern China in 2010, resulting in the death of more than one million piglets [11,12,13]. Subsequently, a variant PEDV strain emerged in the United States in 2013 [14,15,16,17] and rapidly disseminated in North America and globally [18,19], causing significant economic losses.

Based on phylogenetic analysis of the *S* gene, global PEDV strains are classified into two major groups, G1 (classical strains) and G2 (variant strains), with further subdivision into G2a, G2b, and the emerging G2c subgroup [1]. In China, G2a and G2b strains have been predominant; however, since 2020, G2c strains have increasingly been reported [4,20,21]. Although vaccines derived from representative G2 strains (e.g., AJ1102) have contributed to disease control, their protective efficacy against emerging G2c variants remains limited [22,23,24,25]. The rapid evolution and increasing prevalence of G2c PEDV raise concerns regarding viral pathogenicity, immune evasion, and vaccine effectiveness, highlighting the urgent need for further molecular and functional characterization. Therefore, in-depth studies on emerging G2c strains are particularly important.

Reverse genetics systems have significantly advanced the study of viral gene function, pathogenesis, and vaccine development [26]. However, the large genome size of coronaviruses presents substantial challenges for the construction of full-length infectious clones [27]. The yeast artificial chromosome-bacterial artificial chromosome (YAC-BAC) system provides a robust platform for assembling large viral genomes and enabling stable recovery of recombinant viruses [28]. In addition, transformation-associated recombination (TAR) cloning enables rapid and efficient assembly of full-length infectious clones in yeast, substantially shortening the time required for viral genome reconstruction [28,29,30]. Combined with CRISPR/Cas9-mediated genome editing, this system enables precise manipulation of viral genomes, including site-directed mutagenesis, gene deletion, and gene replacement, thereby facilitating functional studies and rational vaccine design [28,31,32].

In this study, a highly virulent PEDV strain was isolated from clinical samples collected in Guizhou Province, China, and was identified as a member of the emerging G2c subgroup based on *S* gene phylogenetic analysis. Using the TAR-based reverse genetics system, a full-length infectious cDNA clone was rapidly constructed, and both recombinant PEDV and an EGFP-expressing reporter virus were successfully rescued. The rescued viruses retained infectivity in vitro and pathogenicity in vivo, and rPEDV-EGFP maintained stable reporter expression during serial passage. These results demonstrate the robustness of the reverse genetics platform established in this study and provide a useful tool for investigating PEDV biology and developing rational vaccine strategies.

## 2. Materials and Methods

### 2.1. Cells, Bacterial and Yeast Strains

Vero CCL-81 and BHK-21 cells were cultured in Dulbecco’s modified Eagle’s medium (DMEM) (Gibco, Waltham, MA, USA) supplemented with 1% penicillin/streptomycin and 10% fetal bovine serum (FBS) (Vazyme, Nanjing, China). Cells were maintained at 37 °C in a humidified incubator with 5% CO_2_. Electrocompetent *Escherichia coli* TOP10 cells and *Saccharomyces cerevisiae* MaV203 competent cells were purchased from Thermo Fisher Scientific (Waltham, MA, USA).

### 2.2. Viral Isolation and Identification

PEDV was isolated from intestinal samples of diarrheic piglets collected during a 2023 outbreak at a pig farm in Bijie, Guizhou Province, China. The virus was propagated in Vero CCL-81 cells cultured in serum-free medium supplemented with 10 µg/mL trypsin (Gibco, Waltham, MA, USA). Confluent Vero CCL-81 cells in 24-well plates were washed twice with phosphate-buffered saline (PBS) and inoculated with a filtered and sterilized homogenized tissue suspension diluted in infection medium. After adsorption at 37 °C with 5% CO_2_ for 2 h, the inoculum was replaced with fresh infection medium, and the cells were further incubated under the same conditions. When >70% of cells exhibited cytopathic effects (CPE), cultures were subjected to three freeze–thaw cycles to release the virus. The harvested virus was either passaged or stored at −80 °C.

The clinical PEDV isolate underwent three rounds of plaque purification, and the clone exhibiting stable CPE was selected as the purified strain. After ten serial passages in Vero CCL-81 cells, the viral genome was sequenced. Viral RNA was extracted and detected by reverse transcription PCR (RT-PCR) using PEDV *S* gene-specific primers (forward, 5′-GAATGGTAAGTTGCTAGTGCG-3′; reverse, 5′-CATTGAGCTCCAACTCTTGG-3′). The isolate was further characterized by indirect immunofluorescence assay (IFA) using a PEDV N-specific monoclonal antibody and by transmission electron microscopy (TEM) to examine viral particle morphology. The isolated strain was designated PEDV-BJ-2023.

### 2.3. Genome Sequencing

Based on conserved regions identified among PEDV strains deposited in the NCBI database, seven pairs of primers (Table 1) were designed to amplify overlapping fragments of the viral genome. First-strand cDNA was synthesized using primer PEDV-R7 and the SuperScript III First-Strand Synthesis System (Thermo Fisher Scientific, Waltham, MA, USA). The resulting cDNA served as a template for PCR amplification of the seven overlapping fragments using Q5 High-Fidelity DNA Polymerase (NEB, Ipswich, MA, USA). PCR products were purified with the Zymoclean Large Fragment DNA Recovery Kit (Zymo Research, Irvine, CA, USA) and subjected to Sanger sequencing (GENEWIZ, Suzhou, China). Sequencing data were assembled and analyzed using SnapGene 7.0.3 software to generate the complete genome sequence of PEDV-BJ-2023. The complete genome sequence was deposited in the GenBank database (https://www.ncbi.nlm.nih.gov/genbank/, accessed on 3 August 2026).

### 2.4. Phylogenetic Analysis

All available PEDV *S* gene and complete genome sequences were retrieved from the NCBI nucleotide database. To reduce redundancy while preserving representative diversity, sequences sharing >95% nucleotide identity were filtered using CD-HIT. Multiple sequence alignments were performed with MAFFT v7 using default parameters. Phylogenetic trees were constructed in MEGA12 using the neighbor-joining (NJ) method, and evolutionary distances were estimated with the Maximum Composite Likelihood model. The robustness of phylogenetic inference was assessed with 1000 bootstrap replicates, and bootstrap support values were indicated at the corresponding nodes. The resulting phylogenetic trees were pruned, re-rooted, and visualized using Interactive Tree Of Life (iTOL, https://itol.embl.de/, accessed on 3 August 2026).

### 2.5. Generation of a Full-Length Infectious cDNA Clone of PEDV

The pYES1L vector was engineered to contain the CMV early promoter, hepatitis delta virus (HDV) ribozyme sequence, and bovine growth hormone (BGH) termination signal. Following the manufacturer’s instructions for the GeneArt High-Order Genetic Assembly System (Thermo Fisher Scientific, Waltham, MA, USA), 100 ng of linearized pYES1L vector and 200 ng of each DNA fragment (F1–F7) were co-transformed into *S. cerevisiae* MaV203 competent cells using a PEG/LiAc solution. The linearized pYES1L vector was amplified with primers pYES1L-F and pYES1L-R, and the viral cDNA template was amplified into seven overlapping fragments (F1–F7) using primer pairs pYES1L-F1/R1, F2/R2, F3/R3, F4/R4, F5/R5, F6/R6, and F7/R7 (Table 2). All PCR amplifications were performed using Q5 High-Fidelity DNA Polymerase (NEB, Ipswich, MA, USA). Each adjacent fragment shared more than 30 nucleotides of homologous overlap to facilitate TAR.

Recombinant yeast colonies were screened by colony PCR using the primers listed in Table 2. Positive clones were lysed, and plasmid DNA was subsequently electroporated into *E. coli* TOP10 cells. Recombinant plasmids were purified using a NucleoBond Xtra Midi Kit (MACHEREY-NAGEL, Düren, Germany), and the integrity of the full-length cDNA clone was verified by sequencing. The resulting infectious clone was designated pYES1L-PEDV-BJ-2023 and used for subsequent virus rescue experiments.

### 2.6. Construction of the Infectious Clone pYES1L-PEDV-EGFP Using the CRISPR/Cas9 System

A cDNA clone carrying an EGFP reporter was generated by inserting the *EGFP* gene at the 5′ end of the *N* gene in the pYES1L-PEDV-BJ-2023 plasmid using the CRISPR/Cas9 system. A single-guide RNA (PEDV-sgRNA; TAGTCTAAACAGAAACTTTA) targeting the 5′ region of the *N* gene was designed and complexed with Cas9 nuclease (NEB, Ipswich, MA, USA). The sgRNA-Cas9 complex was incubated with 3 µg of pYES1L-PEDV-BJ-2023 plasmid to mediate site-specific cleavage following the manufacturer’s instructions. The linearized plasmid was confirmed by electrophoresis and purified using the Zymoclean Large Fragment DNA Recovery Kit (Zymo Research, Irvine, CA, USA).

In parallel, the *EGFP* sequence was amplified together with a T2A peptide linker, generating a fragment with ~20 bp homology arms corresponding to the insertion site. The fragment was assembled into the linearized vector using the HiFi DNA Assembly Master Mix (NEB, Ipswich, MA, USA). The resulting recombinant construct was designated pYES1L-PEDV-EGFP.

### 2.7. Rescue and Characterization of Recombinant PEDV

Infectious clone plasmids were transfected into BHK-21 cells using Lipofectamine 3000 transfection reagent (Invitrogen, Carlsbad, CA, USA) according to the manufacturer’s instructions. Briefly, BHK-21 cells were seeded in 6-well plates and transfected with 2 µg of either pYES1L-PEDV-BJ-2023 or pYES1L-PEDV-EGFP plasmid when cell confluence reached ~80%. At 48 h post-transfection (hpt), culture supernatants containing virus particles were collected. A portion of the supernatant was stored at −80 °C, while the remainder was used to infect Vero CCL-81 cells seeded in 24-well plates at >80% confluence. After a 2 h adsorption period, the inoculum was replaced with infection medium, and cells were maintained at 37 °C in 5% CO_2_. CPE or EGFP fluorescence was monitored daily. After approximately 5 days, infected cells and supernatants were harvested and stored at −80 °C for subsequent passage in Vero CCL-81 cells.

### 2.8. IFA

Vero CCL-81 cells were seeded onto sterile glass coverslips in 24-well plates and infected with PEDV at ~80% confluence. At the indicated time points, cells were fixed with 4% paraformaldehyde at room temperature for 15 min and air-dried. Fixed cells were permeabilized with 0.01% Triton X-100 for 10 min at room temperature and blocked with 1% bovine serum albumin (BSA) for 1 h. Cells were then incubated overnight at 4 °C with mouse anti-PEDV N monoclonal antibody and/or rabbit anti-GFP monoclonal antibody (ET1607-31, HUABIO, Hangzhou, China), diluted in PBS. After three washes with PBS, cells were incubated with the appropriate Alexa Fluor 488- or Alexa Fluor 594-conjugated secondary antibodies against mouse or rabbit IgG (H+L) (Jackson ImmunoResearch, West Grove, PA, USA) at 37 °C for 1 h; the cells were then washed three times with PBS and stained with 4′,6-diamidino-2-phenylindole (DAPI) (Beyotime, Shanghai, China) for 5 min at room temperature. Coverslips were mounted with antifade mounting medium, and images were captured using an Olympus BX63 fluorescence microscope (Olympus, Tokyo, Japan).

### 2.9. Western Blot

Vero CCL-81 cells were infected with virus at ~80% confluence. At the indicated time points post-infection, culture medium was removed and cells were washed with PBS. Total cellular proteins were extracted using WB and IP lysis buffer (Biosharp, Hefei, China) supplemented with protease inhibitors (MCE, Monmouth Junction, NJ, USA). Protein samples were mixed with 5× loading buffer and denatured at 100 °C for 5 min. Proteins were separated by 12% SDS-PAGE and transferred onto nitrocellulose membranes using a wet transfer system. Membranes were blocked with 5% non-fat dry milk at room temperature for 1 h, followed by incubation overnight at 4 °C with primary antibodies. After five washes with PBST, membranes were incubated with HRP-conjugated goat anti-mouse IgG (Cat# D110087, Sangon Biotech, Shanghai, China) or HRP-conjugated goat anti-rabbit IgG (Cat# D110058, Sangon Biotech, Shanghai, China) at room temperature for 1 h, followed by five additional washes with PBST. The proteins were visualized using an enhanced chemiluminescence (ECL) substrate and imaged with the SH-Cute 523 chemiluminescence imaging system (SHST, Hangzhou, China).

### 2.10. RNA Extraction and RT-qPCR

Viral RNA was extracted using the Viral RNA Extraction Kit (TIANGEN, Beijing, China), and total RNA from animal tissues was extracted using RNA isolater Total RNA Extraction Reagent (Vazyme, Nanjing, China), following the manufacturers’ instructions. RNA was eluted in RNase-free water and quantified using a NanoDrop OneC spectrophotometer (Thermo Fisher Scientific, Waltham, MA, USA). RT-qPCR was performed using the HiScript II One Step qRT-PCR Probe Kit (Vazyme, Nanjing, China) with specific primers and a TaqMan probe (PEDV-N-qF: 5′-CCCACTAACCTGGGTGTCAGA-3′; PEDV-N-qR: 5′-GCTGGGAAGCTGTTGAGAGAA-3′; PEDV-N-Probe: 5′-FAM-AGGCGTCTGAAAAGCCAATTATTCCA-BHQ1-3′). Amplification efficiency and linearity were evaluated using the standard plasmid dilutions. The resulting standard curve (*Y* = −3.3523*X* + 39.643, *R*^2^ = 0.9998) indicated a strong linear correlation, and viral RNA copy numbers in samples were calculated based on Ct values relative to this curve.

### 2.11. Viral Growth Curves

Vero CCL-81 cells in 24-well plates were infected with PEDV at a multiplicity of infection (MOI) of 0.1. After 2 h of adsorption, cells were washed and maintained in infection medium. Cells and supernatants were collected at 0, 12, 24, 36, 48, and 60 hpi, with three biological replicates per time point. Viral titers were determined by the 50% tissue culture infectious dose (TCID_50_) assay, and growth curves were plotted using GraphPad Prism 10.

### 2.12. Plaque Assay

Vero CCL-81 cells were seeded in 12-well plates and cultured to full confluence. Tenfold serial dilutions of PEDV were added to the monolayers and allowed to adsorb for 2 h at 37 °C. After removing the inoculum and washing twice with PBS, cells were overlaid with DMEM containing 1% UltraPure™ Low Melting Point Agarose (Thermo Fisher Scientific, Waltham, MA, USA) and 10 µg/mL trypsin. At 3 days post-infection, cells were fixed with 4% paraformaldehyde for 15 min at room temperature and stained with 0.1% crystal violet for plaque visualization.

### 2.13. Purification of PEDV Particles by Ultracentrifugation

Clarified culture supernatants containing PEDV were layered onto 20% (*w*/*v*) sucrose cushions in PBS and ultracentrifuged at 100,000× *g* for 2 h at 4 °C using a Beckman Coulter ultracentrifuge (Beckman Coulter, Brea, CA, USA) with a Type 70 Ti rotor. Viral pellets were gently resuspended in cold PBS for TEM analysis.

### 2.14. Animal Experiments

A total of 12 five-day-old clinically healthy piglets were obtained from a PEDV-free commercial farm and acclimated for 1 day. All piglets were confirmed negative for ASFV, CSFV, PRRSV, PEDV, PDCoV, TGEV, PoRV, and JEV by PCR prior to the experiment. Piglets were randomly assigned to four groups and orally inoculated as follows: Group 1, PEDV-BJ-2023 (2 × 10^5^ PFU per piglet); Group 2, rPEDV (2 × 10^5^ PFU per piglet); Group 3, rPEDV-EGFP (2 × 10^5^ PFU per piglet); Group 4, DMEM control (equal volume). Each group was housed separately under controlled environmental conditions (25 °C, 50% humidity).

After inoculation, piglets were monitored every 12 h for clinical signs, including diarrhea, vomiting, and general condition. Clinical signs were scored according to the criteria shown in Table 3. Body weights were recorded immediately prior to necropsy and compared with pre-inoculation weights. Nasal and rectal swabs were collected daily to assess viral shedding.

### 2.15. Histopathology and Immunohistochemistry

To assess the acute-phase pathogenicity of PEDV strains, piglets were euthanized at 36 hpi, and samples from the duodenum, jejunum, ileum, cecum, and colon were collected and fixed in 4% paraformaldehyde for 48 h. Tissues were processed for hematoxylin and eosin (H&E) staining or immunohistochemistry (IHC) using a PEDV N-specific monoclonal antibody. Histopathological analysis included measurement of the villus height-to-crypt depth (VH:CD) ratio in the jejunum. Intestinal samples collected at necropsy were also used to quantify viral loads in different segments by RT-qPCR.

### 2.16. Statistical Analysis

Statistical analyses were performed using GraphPad Prism 10. Differences between two groups were assessed by unpaired Student’s *t*-test, and comparisons among multiple groups were analyzed by one-way ANOVA with Tukey’s post hoc test. Data are presented as mean ± SD, and *p* < 0.05 was considered statistically significant.

## 3. Results

### 3.1. Isolation and Identification of a PEDV Field Strain

**Figure 1:**
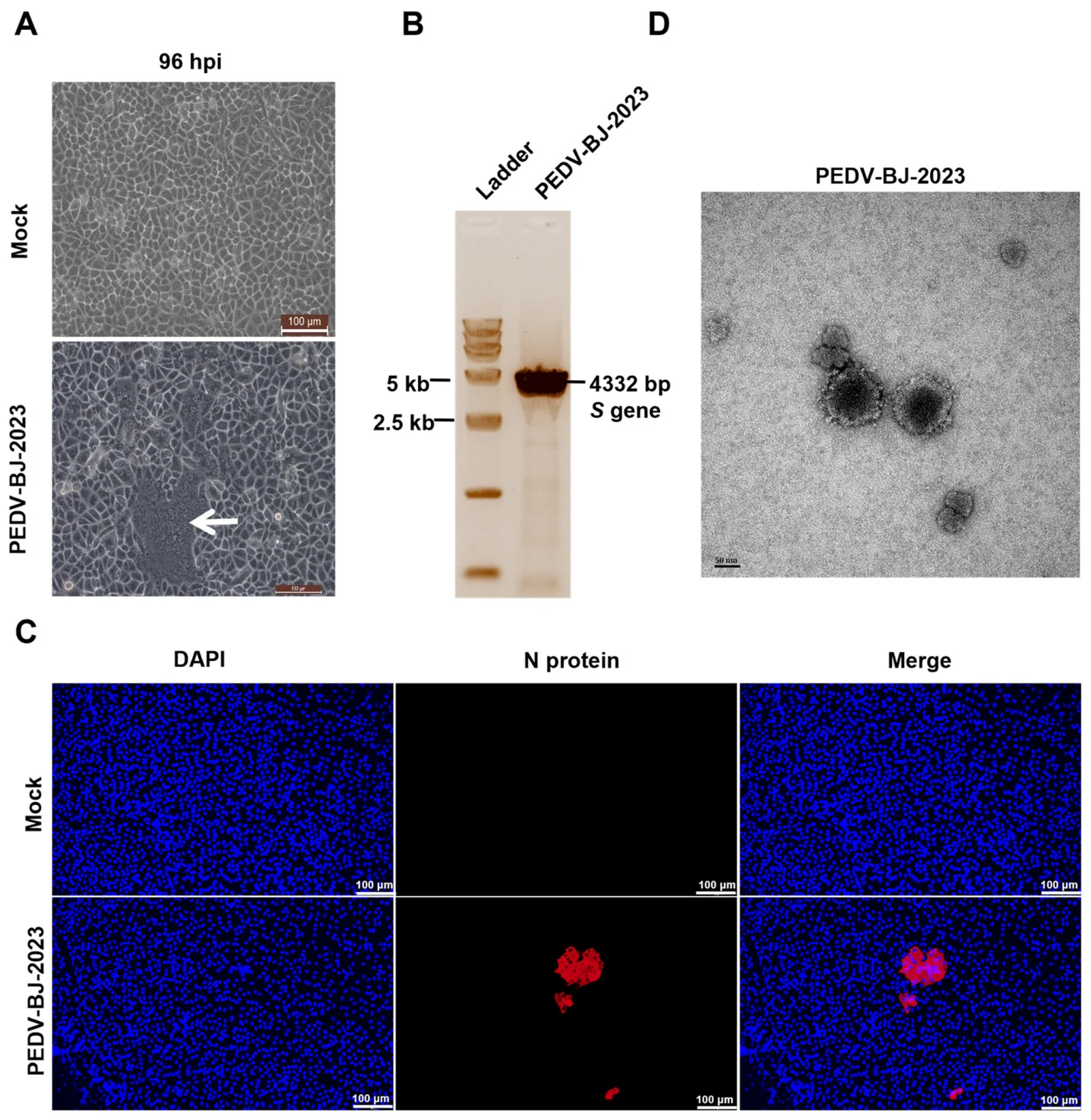
Viral isolation and identification. (**A**) Vero CCL-81 cells inoculated with the clinical sample and observed at 96 hpi by bright-field microscopy; white arrows indicate syncytia. (**B**) RT-PCR amplification of the PEDV *S* gene from viral supernatants; M, DL15000 DNA marker (Vazyme, Nanjing, China). (**C**) Detection of PEDV N protein expression by indirect immunofluorescence assay; PEDV N protein is shown in red, and nuclei were counterstained with DAPI (blue). (**D**) Virus particles purified from the supernatant of the PEDV strain and visualized by TEM.

In this study, intestinal tissue samples were collected from piglets that had died following severe diarrhea during an outbreak in Bijie City, Guizhou, China. After processing, the samples were inoculated into Vero CCL-81 cells, and syncytial formation was observed in infected cells at 4 dpi, while no such formation occurred in the control group (Figure 1A). RT-PCR using PEDV *S* gene-specific primers yielded a positive band of ~4.3 kb (Figure 1B). IFA with a PEDV N protein-specific antibody showed cytoplasmic fluorescence in infected cells, confirming viral infection (Figure 1C). As shown in Figure 1D, TEM revealed viral particles with characteristic crown-like projections, 80–120 nm in diameter, consistent with coronaviruses. Collectively, these results demonstrate the successful isolation and identification of a PEDV strain from the clinical samples.

### 3.2. PEDV-BJ-2023 Belongs to the Recently Emerged G2c Genotype

**Figure 2:**
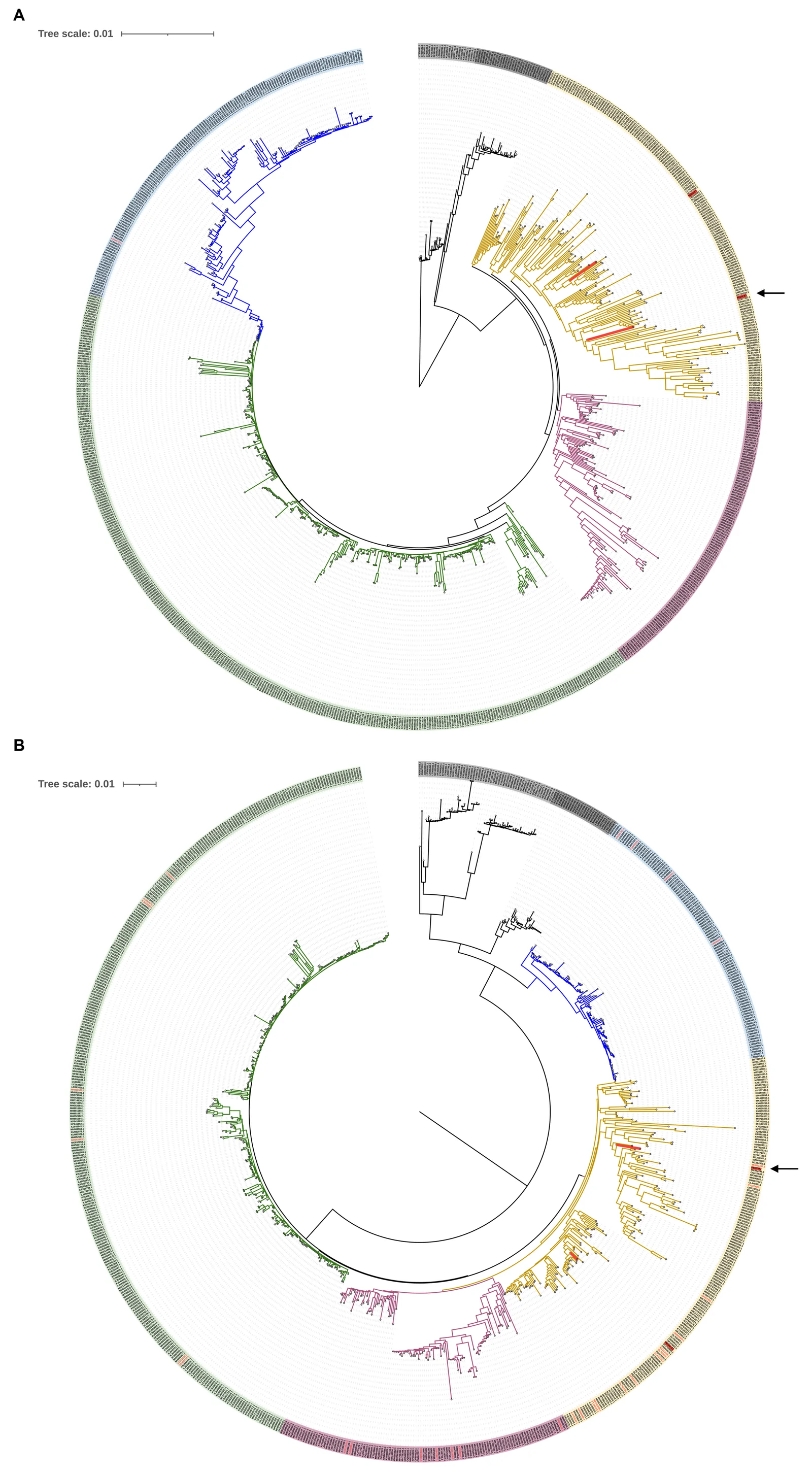
Phylogenetic analysis of PEDV strains. (**A**) Phylogenetic analysis based on the complete genome of PEDV. (**B**) Phylogenetic analysis based on the *S* gene. PEDV-BJ-2023 is highlighted in red and indicated by black arrows. Distinct genetic subgroups are indicated by colors in the phylogenetic trees: G1a (light gray), G1b (dark gray), G2a (green), G2b (magenta), G2c (orange), and S-Indel (light blue).

For genomic characterization of the PEDV strain identified in this study, its complete genome sequence, designated PEDV-BJ-2023, was determined and deposited in the GenBank database under accession number PV098397. Phylogenetic analyses based on both the complete genome and the *S* gene were performed using datasets comprising over 800 reference sequences each. The results consistently placed PEDV-BJ-2023 within the recently emerging G2c lineage (Figure 2).

### 3.3. Generation of an Infectious cDNA Clone and Recovery of Recombinant PEDV-BJ-2023

**Figure 3:**
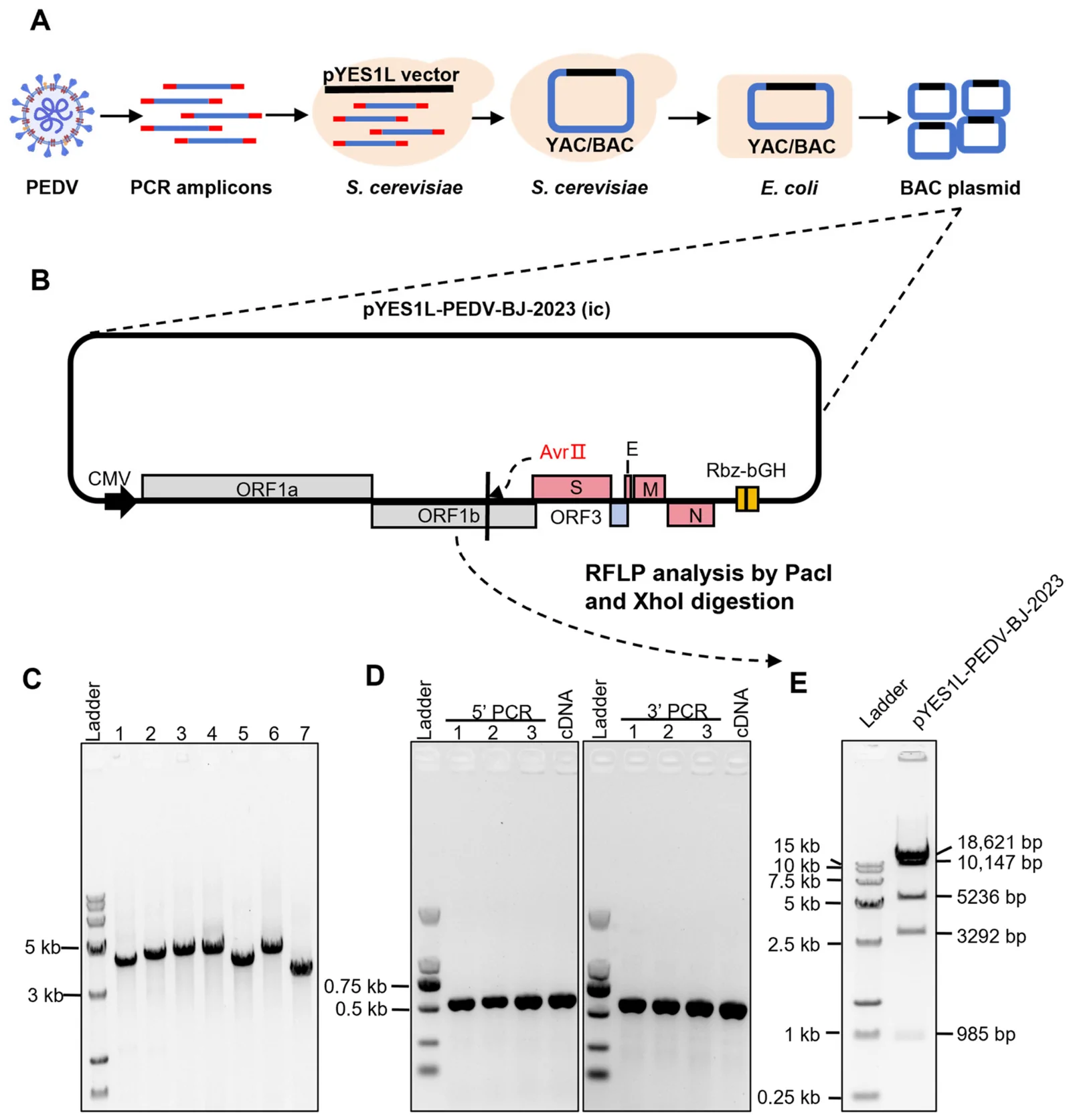
Construction of infectious cDNA clone pYES1L-PEDV-BJ-2023 by homologous recombination in yeast. (**A**) PEDV infectious cDNA clone assembly procedure. (**B**) Schematic diagram of pYES1L-PEDV-BJ-2023. (**C**) Agarose gel electrophoresis of seven overlapping PEDV-BJ-2023 cDNA fragments (F1–F7); M, Trans 15K DNA Marker (TransGen, Beijing, China). (**D**) Colony PCR verification of recombinant yeast clones; lanes 1–3: different yeast colonies; lane cDNA: PEDV-BJ-2023 cDNA generated by reverse transcription (positive control); M, Trans 2K DNA marker (TransGen, Beijing, China). Primer pairs cPCR-1 and cPCR-2 amplified the expected fragments from recombinant clones. (**E**) Restriction fragment length polymorphism (RFLP) analysis. PacI and XhoI digestion of pYES1L-PEDV-BJ-2023 yielded five fragments (18,621, 10,147, 5236, 3292, and 985 bp) on a 0.8% agarose gel, consistent with the expected sizes; M, Trans 15K DNA marker (TransGen, Beijing, China).

Given the large genome size of PEDV-BJ-2023 (~28 kb), the pYES1L BAC/YAC shuttle vector was selected as the backbone for full-length cDNA assembly. Prior to assembly, a CMV early promoter, HDV ribozyme sequence, and BGH terminator were inserted into pYES1L to drive efficient genome expression. The viral cDNA was divided into seven overlapping fragments and assembled in *S. cerevisiae* via homologous recombination. Two synonymous substitutions were introduced into *Nsp13* to disrupt the AvrII restriction site for differentiation from the wild-type strain (Figure 3A–C). After three days of incubation on selective medium, colony PCR of three randomly selected yeast clones confirmed the expected 5′ and 3′ junctions (Figure 3D). One verified clone was transformed into *E. coli* for propagation and plasmid amplification. Restriction enzyme digestion with PacI and XhoI yielded the expected fragment pattern (Figure 3E), and the integrity of the infectious clone, designated pYES1L-PEDV-BJ-2023, was further verified by full-length sequencing.

**Figure 4:**
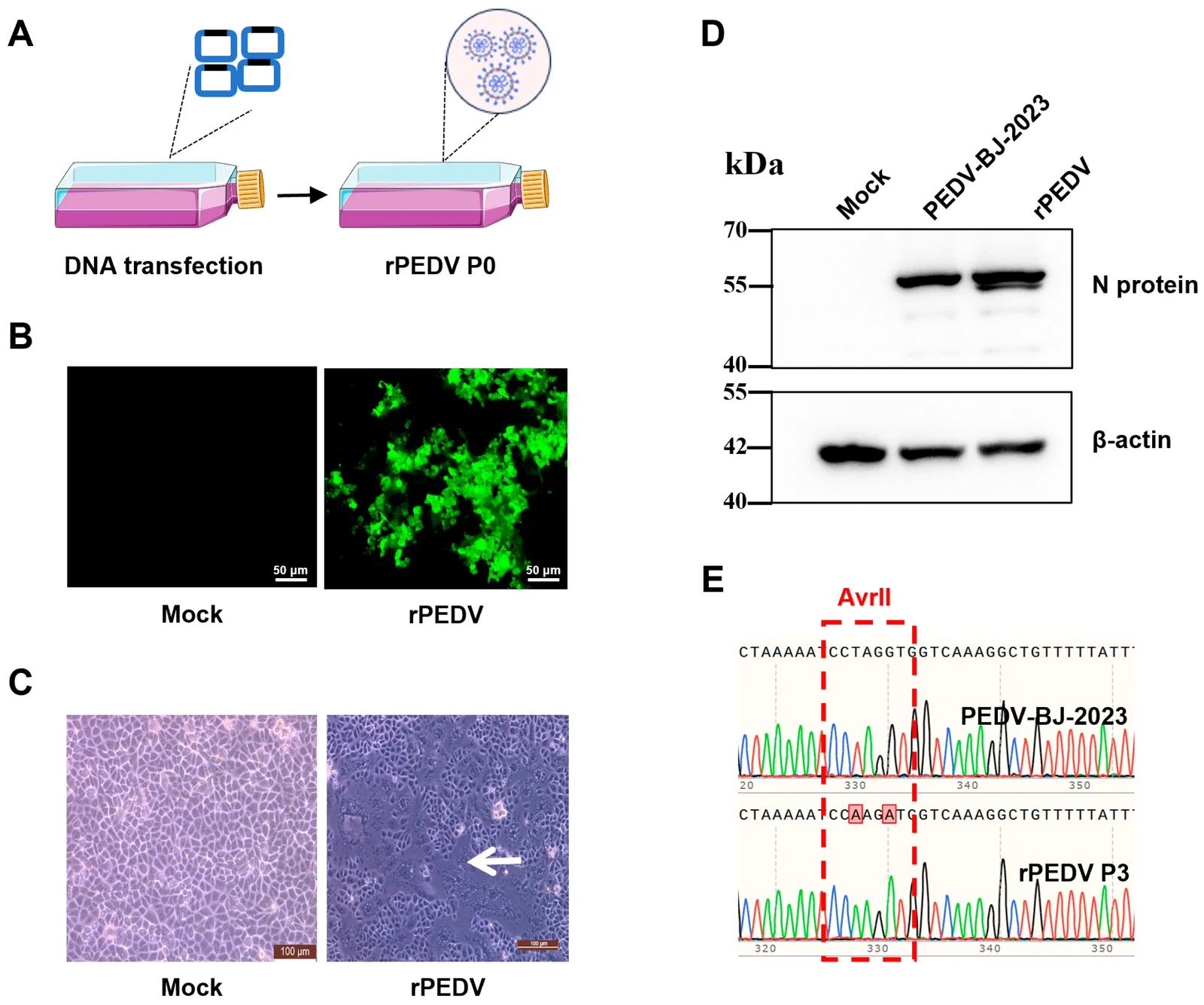
Recovery and verification of recombinant PEDV. (**A**) Schematic diagram of recombinant PEDV rescue. (**B**) Confirmation of recombinant virus rescue in BHK-21 cells by immunofluorescence assay; PEDV N protein is shown in green. (**C**) CPE was observed in Vero CCL-81 cells under a light microscope at 48 hpi with the recombinant virus; white arrows indicate syncytia. (**D**) Detection of N protein expression in Vero CCL-81 cells infected with rPEDV by Western blot. (**E**) Sanger sequencing confirmed the presence of the designed synonymous substitutions in the recombinant PEDV genome; the designed synonymous substitutions are highlighted by red shaded boxes.

As shown in Figure 4A, the recombinant virus was rescued by transfecting the infectious clone pYES1L-PEDV-BJ-2023 into BHK-21 cells. An overexpression plasmid encoding the PEDV-BJ-2023 N protein was optionally co-transfected to enhance the rescue efficiency. The culture supernatant containing recombinant viral particles was subsequently used to infect Vero CCL-81 cells. BHK-21 cells transfected with the infectious clone alone were fixed for IFA to verify successful rescue (Figure 4B). At 48 hpi, Vero CCL-81 cells exhibited characteristic syncytium formation (Figure 4C), and N protein expression was confirmed by Western blot (Figure 4D). The recombinant virus was further passaged, and viral RNA from the third passage was subjected to RT-PCR amplification of the genetically marked region. Sequencing of the PCR product confirmed the presence of the designed synonymous mutations (Figure 4E), verifying the successful rescue of rPEDV.

### 3.4. Engineering of an EGFP-Reporter PEDV for Direct Visualization of Viral Infection

**Figure 5:**
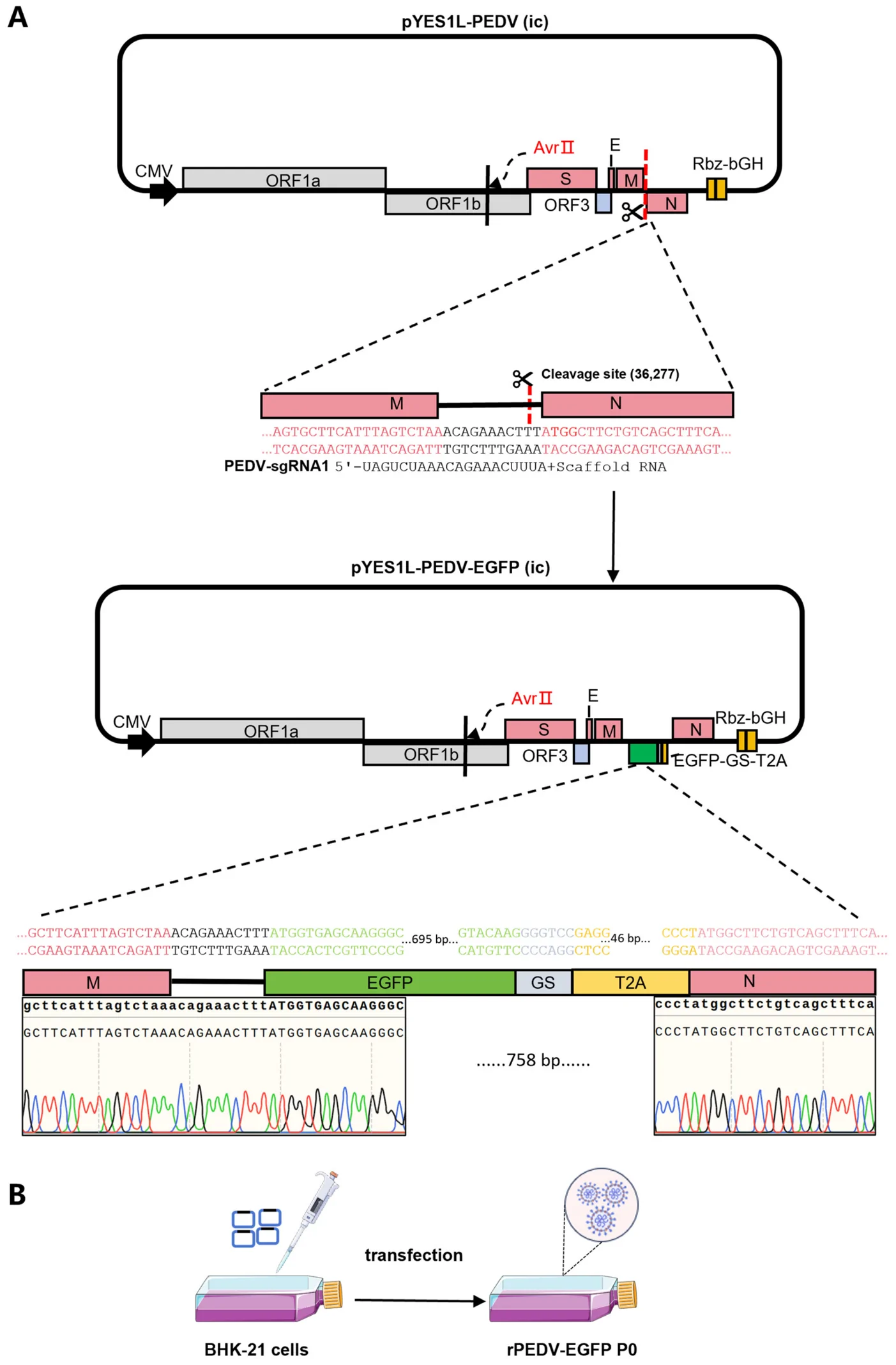
Schematic overview of rPEDV-EGFP construction and rescue. (**A**) Schematic diagram of the construction strategy and map of pYES1L-PEDV-EGFP. (**B**) Schematic diagram of the recombinant fluorescent PEDV rescue.

A fluorescent reporter virus was generated by inserting the *EGFP* gene immediately upstream of the *N* gene, linked via a self-cleaving 2A peptide. To enable rapid and efficient assembly of the infectious clone, a single-guide RNA was designed to target the 5′ region of the *N* gene within pYES1L-PEDV-BJ-2023. Cas9 nuclease introduced a precise double-strand break at this site, facilitating site-specific insertion of the EGFP-2A fragment via homologous recombination (Figure 5A). The recombinant plasmid was electroporated into *E. coli* TOP10 competent cells, and positive colonies were identified by colony PCR before subsequent experiments. The verified infectious clone, designated pYES1L-PEDV-EGFP, was then used for virus rescue following the same procedure as for the parental clone (Figure 5B).

**Figure 6:**
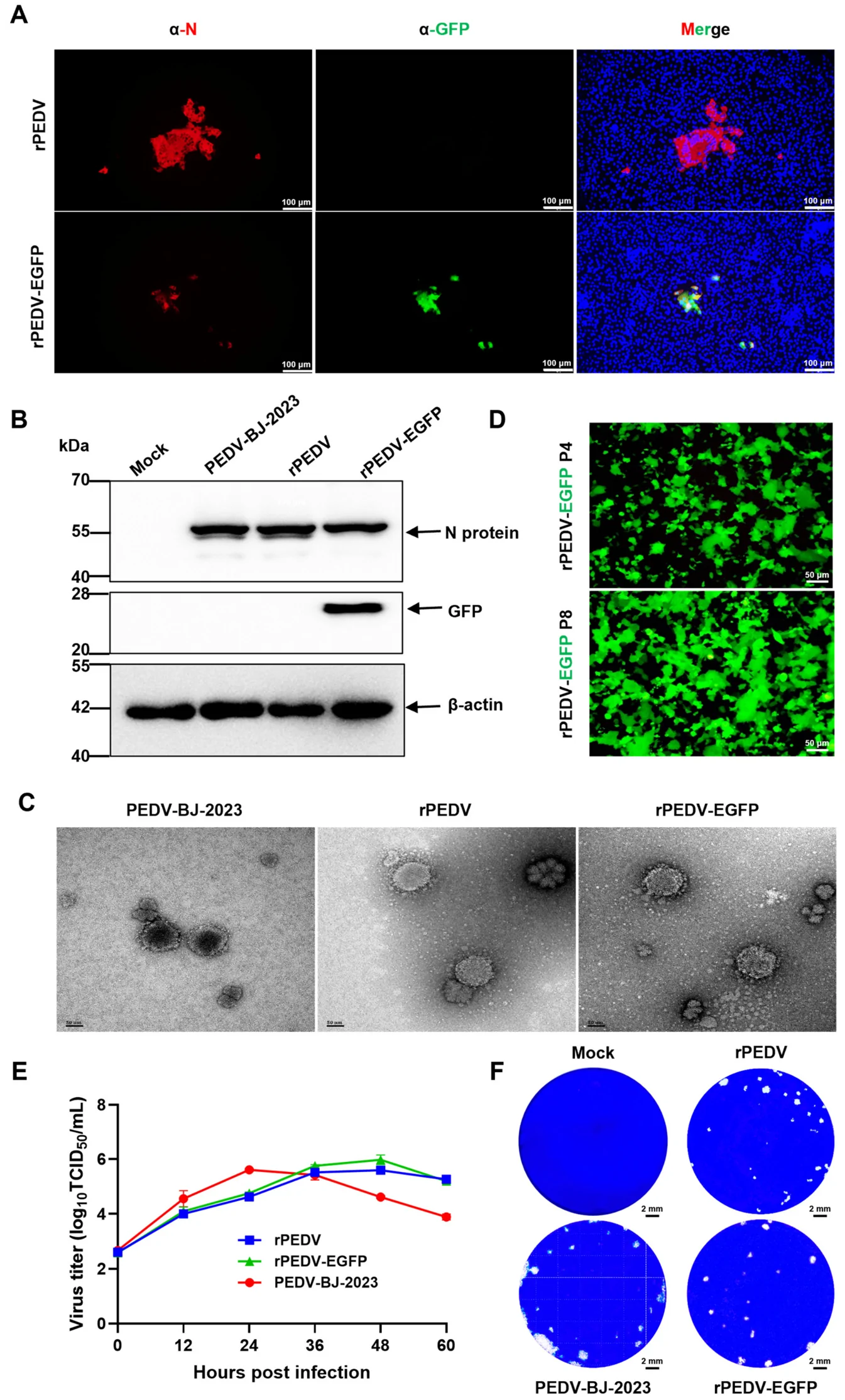
Verification and comparative biological characterization of the rPEDV-EGFP reporter virus. (**A**) Immunofluorescence of Vero CCL-81 cells infected with rPEDV or rPEDV-EGFP, stained with anti-PEDV N and anti-GFP antibodies (ET1607-31, HUABIO, Hangzhou, China); PEDV N protein is shown in red, EGFP in green, and nuclei were counterstained with DAPI (blue). (**B**) Identification of rPEDV-EGFP in Vero CCL-81 cells by Western blot. (**C**) Negative-staining TEM of three types of PEDV virions. (**D**) Fluorescence images of Vero CCL-81 cells infected with rPEDV-EGFP (MOI = 0.1) at different passages. EGFP fluorescence was monitored at 24 hpi to assess the stability of reporter expression during serial passage. (**E**) Multi-step growth kinetics of PEDV in Vero CCL-81 cells at MOI of 0.1. (**F**) Plaque morphology of PEDV-BJ-2023, rPEDV, and rPEDV-EGFP.

At 24 hpt, distinct EGFP fluorescence was detected in BHK-21 cells transfected with the recombinant plasmid, confirming successful expression of the inserted reporter gene. The recombinant virus, designated rPEDV-EGFP, was further characterized. IFA showed that N protein was detected in cells infected with either rPEDV or rPEDV-EGFP, whereas GFP fluorescence was observed only in rPEDV-EGFP-infected cells, with N and GFP signals co-localized (Figure 6A). Consistently, Western blot analysis confirmed N protein expression in cells infected with all three viruses, whereas GFP protein was detected only in rPEDV-EGFP-infected cells, further validating the successful rescue of the recombinant reporter virus (Figure 6B). TEM analysis of purified virions showed spherical or pleomorphic particles with clearly visible spike projections, with no major morphological abnormalities in the recombinant viruses (Figure 6C). Given that fluorescent reporter viruses may exhibit instability during serial passaging, such as loss or attenuation of exogenous gene expression, we systematically evaluated the genetic stability of rPEDV-EGFP. Under identical MOI conditions, both fourth- and eighth-passage viruses produced clear and reproducible EGFP signals, with the eighth passage displaying more uniform and robust fluorescence without any detectable loss or reduction in expression (Figure 6D). These results demonstrate that rPEDV-EGFP maintains stable exogenous gene expression over multiple passages, serving as a reliable fluorescent reporter system for monitoring viral replication, assessing antiviral responses, and investigating virus–host interactions.

After generating and molecularly verifying recombinant rPEDV and rPEDV-EGFP, we compared their biological properties and replication kinetics with the parental wild-type PEDV-BJ-2023 in vitro and in vivo. Notably, the wild-type virus replicated faster during the early phase, causing stronger CPE and substantial host cell death (Figure 6E), which led to a sharp decline in its titers. In contrast, the recombinant viruses replicated more slowly but maintained relatively stable titers. Consequently, while the wild-type virus reached its peak titer at 24 hpi, the recombinant strains achieved comparable titers at 48 hpi. Plaque assays on Vero CCL-81 cells showed that the size and morphology of rPEDV and rPEDV-EGFP plaques were comparable to those of the wild-type virus, with no obvious differences (Figure 6F).

### 3.5. Comparative Pathogenicity of PEDV-BJ-2023, rPEDV, and rPEDV-EGFP in Piglets

**Figure 7:**
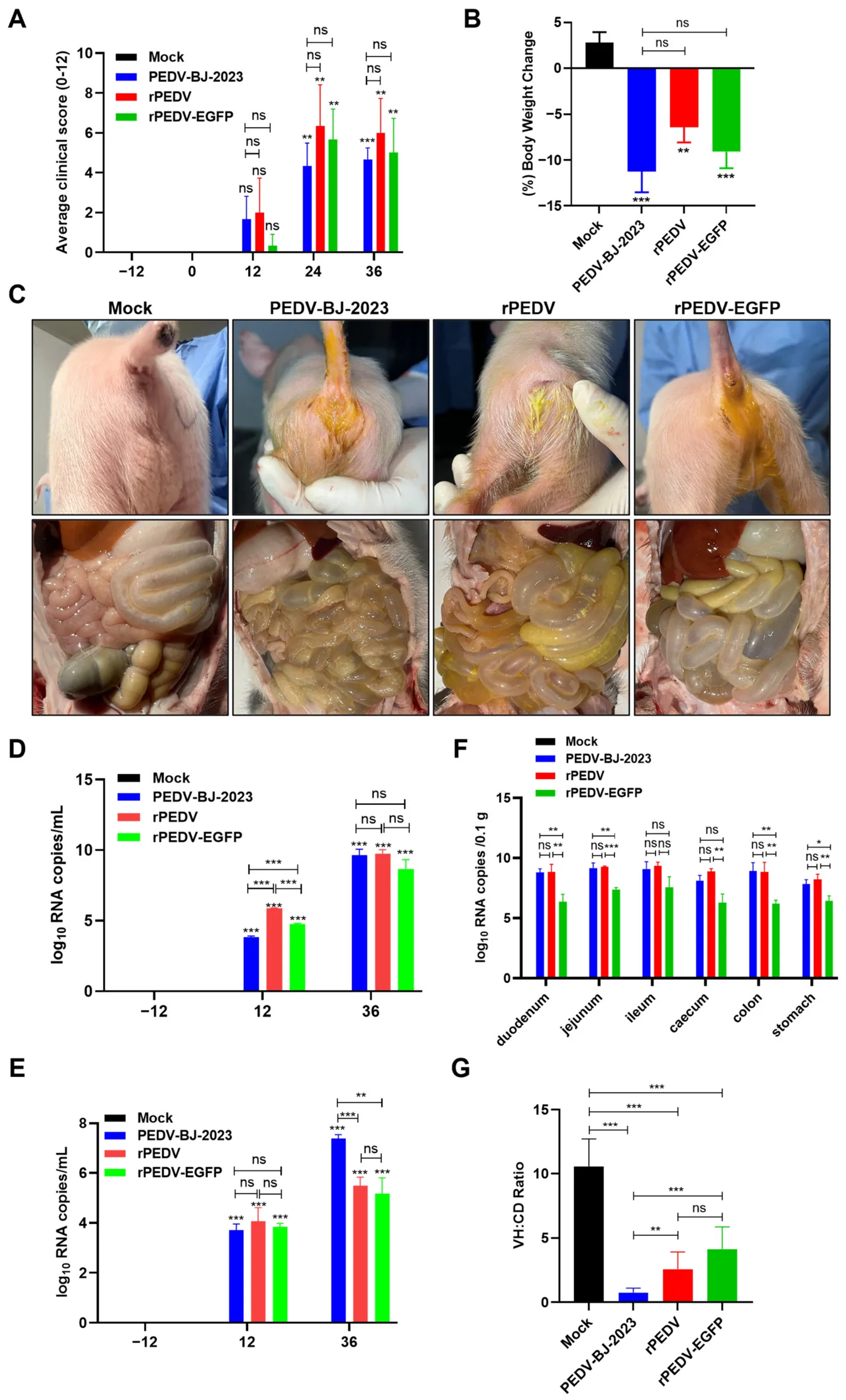
Comparative pathogenicity of parental and recombinant PEDV strains in piglets. (**A**) Clinical scores of diarrhea, activity level, and vomiting. (**B**) Relative body weight changes before inoculation and at 36 hpi. (**C**) Gross anatomy and organ lesions of piglets in the control group inoculated with DMEM and experimental groups inoculated with PEDV-BJ-2023, rPEDV, and rPEDV-EGFP. (**D**) Viral load of rectal swabs. (**E**) Viral load of nasal swabs. (**F**) Viral load of intestinal tissues (duodenum, jejunum, ileum, cecum, colon) and stomach. (**G**) Villus height-to-crypt depth (VH:CD) ratios in the jejunum at 36 hpi. Statistical significance: ns, not significant; *, *p* < 0.05; **, *p* < 0.01; ***, *p* < 0.001.

To further evaluate the pathogenicity of the three PEDV strains, 5-day-old piglets were inoculated. Mild clinical signs were observed in all infected groups at 12 hpi, and by 24 hpi, vomiting and diarrhea were severe (Figure 7A). Piglets in the mock group gained weight compared with pre-inoculation levels, whereas all infected groups exhibited weight loss, with PEDV-BJ-2023-infected piglets showing the greatest decrease (Figure 7B). Necropsy revealed that, compared with the control group, the small intestinal walls of piglets in the PEDV-BJ-2023, rPEDV, and rPEDV-EGFP groups were thinned and translucent, with intestinal distension and large amounts of yellow watery feces (Figure 7C). No lesions were observed in other organs.

Viral shedding in nasal and rectal swabs was assessed at 12 and 36 hpi. All mock pigs remained negative at both time points. In the experimental groups, PEDV RNA was detectable in both nasal and rectal swabs at 12 hpi, with viral loads increasing by 36 hpi. Rectal swabs consistently exhibited higher viral loads than nasal swabs (Figure 7D,E). Viral loads in different intestinal segments and the stomach were further quantified using the TaqMan probe method. PEDV RNA was detected in the duodenum, jejunum, ileum, cecum, colon, and stomach, with the jejunum harboring the highest viral loads (Figure 7F). rPEDV-EGFP exhibited significantly lower viral loads than the other two strains, possibly due to the insertion of the EGFP reporter affecting viral replication.

**Figure 8:**
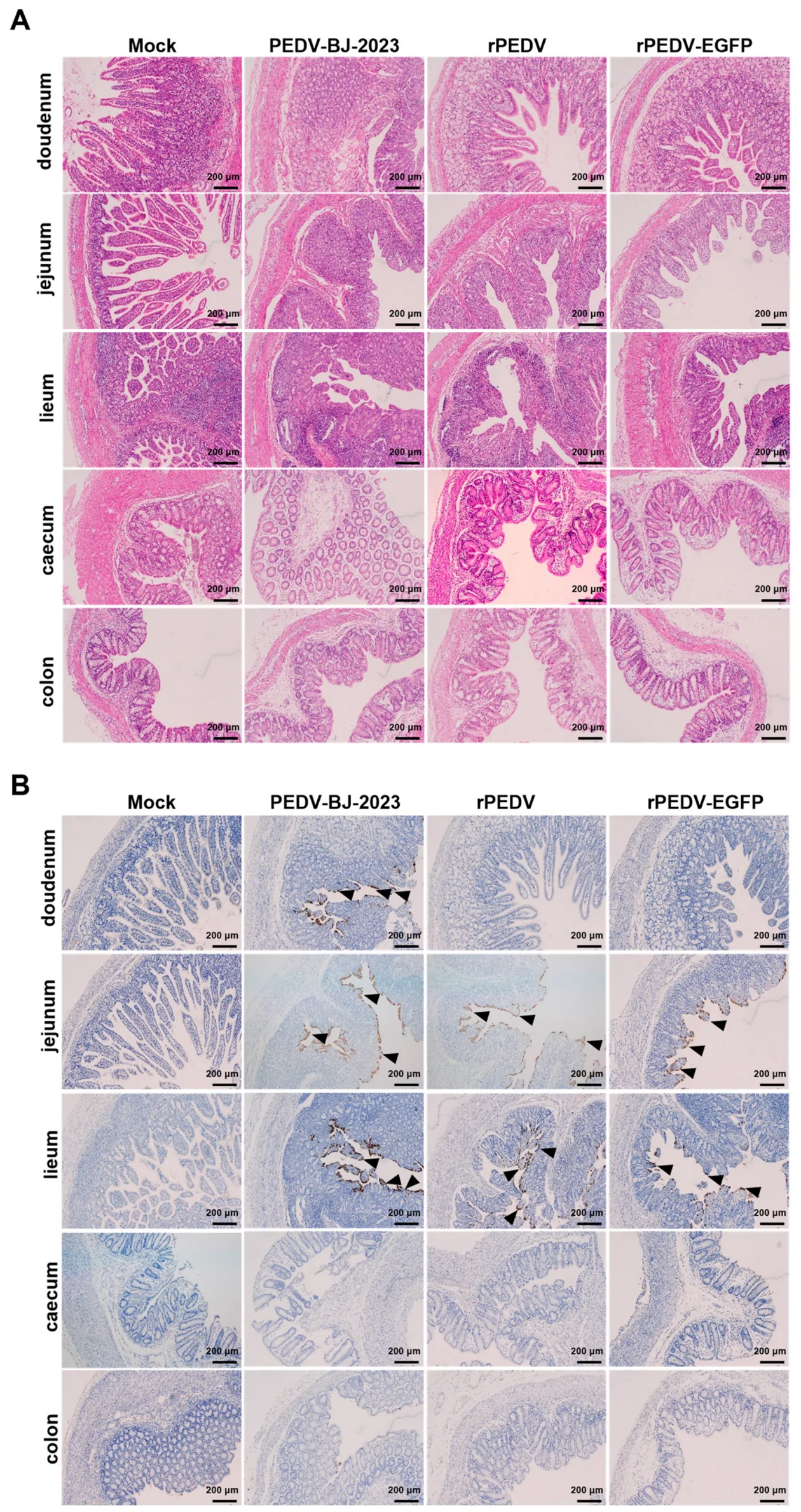
Histopathological analysis of intestinal tissues in piglets. (**A**) Representative H&E-stained sections of the duodenum, jejunum, ileum, cecum, and colon from piglets infected with PEDV-BJ-2023, rPEDV, or rPEDV-EGFP, and from mock-infected controls. (**B**) Representative IHC-stained sections of the duodenum, jejunum, ileum, cecum, and colon from piglets infected with PEDV-BJ-2023, rPEDV, or rPEDV-EGFP, and from mock-infected controls. Black arrows indicate antigen-positive regions.

At 36 hpi, pathological lesions in all PEDV-infected groups were predominantly observed in the jejunum and ileum. H&E staining revealed marked villus atrophy and exfoliation, epithelial necrosis, disruption of the mucosal architecture, and extensive inflammatory cell infiltration in the lamina propria (Figure 8A). In contrast, no evident histopathological alterations were observed in the cecum or colon across the infected groups, which remained comparable to the mock-infected controls. To further assess intestinal damage, the villus height-to-crypt depth (VH:CD) ratio was measured in the jejunum (Figure 7G). All infected groups exhibited a significant reduction in VH:CD ratio in the jejunum, with the lowest ratio observed in the PEDV-BJ-2023 group, followed by the rPEDV group and then the rPEDV-EGFP group.

Immunohistochemistry revealed widespread distribution of PEDV N protein in the jejunum and ileum of infected piglets, with the strongest staining observed in the PEDV-BJ-2023 group (Figure 8B). Notably, PEDV N-positive staining was also detected in the duodenum of PEDV-BJ-2023-infected piglets, whereas no duodenal staining was observed in the other groups. No PEDV N staining was detected in any intestinal segments of the mock-infected controls.

## 4. Discussion

In this study, we isolated a highly virulent PEDV field strain (PEDV-BJ-2023) belonging to the emerging G2c subgroup and established a rapid and efficient reverse genetics platform based on TAR cloning. By integrating yeast-mediated homologous recombination with CRISPR/Cas9-assisted genome editing, we successfully generated a full-length infectious cDNA clone and rescued both rPEDV and the EGFP-expressing reporter virus rPEDV-EGFP. Collectively, these results demonstrate the applicability of TAR-based systems to newly emerging PEDV variants and provide a versatile platform for both mechanistic investigations and translational applications.

The emergence and rapid spread of G2c PEDV variants pose a significant challenge to current control strategies [4,33,34]. Increasing evidence indicates that vaccines derived from earlier G2 strains, such as AJ1102, confer suboptimal protection against G2c viruses [22,25,35]. This reduced efficacy may be associated with ongoing viral evolution, particularly mutations in the S protein, which may alter antigenicity, receptor binding affinity, and immune recognition [36,37,38]. Such changes can facilitate immune escape and sustained transmission in swine populations [39]. Despite these concerns, experimental systems enabling direct functional interrogation of G2c-specific mutations remain limited. The infectious clone established here addresses this gap by providing a tractable system to dissect the molecular determinants of viral evolution, pathogenicity, and immune evasion.

Traditional coronavirus reverse genetics approaches, including BAC-based assembly and in vitro ligation of multiple cDNA fragments [40,41,42], are often constrained by technical complexity, time requirements, and genetic instability [27,43,44]. These limitations are largely attributable to the considerable genome size of coronaviruses (~28 kb) and the presence of sequences that are unstable or toxic in *E. coli* [43,44,45]. In contrast, TAR cloning leverages the highly efficient homologous recombination machinery of yeast to assemble multiple overlapping DNA fragments in a single step [28,46]. Using this strategy, we assembled the complete PEDV genome from seven fragments within approximately one week, thereby substantially accelerating the construction process. Given the considerable economic losses associated with PEDV outbreaks, particularly in intensive swine production systems, the development of a rapid and flexible reverse genetics platform is of clear practical relevance.

Although both recombinant viruses exhibited slightly delayed replication kinetics during the early stage of infection compared with the parental wild-type strain, comparable transient differences in replication kinetics have previously been reported for reverse genetics-derived recombinant coronaviruses [47,48]. The underlying mechanism remains unclear. The two synonymous substitutions introduced into *Nsp13* may influence codon usage, local RNA secondary structure, or other regulatory features. However, these possibilities were not experimentally evaluated in the present study; therefore, no causal relationship can be established. Further studies are required to clarify the molecular basis of this transient replication delay.

An additional strength of this system is its capacity for precise genome manipulation, as demonstrated by the successful insertion of an *EGFP* reporter gene using CRISPR/Cas9. Unlike previously reported strategies in which the *ORF3* gene was replaced with reporter sequences [32,46,49], we inserted the EGFP-2A cassette into the M-N genomic region while preserving the complete viral coding repertoire. This strategy was designed to minimize disruption of viral genetic elements, particularly *ORF3*, which has been suggested to contribute to viral fitness and host–virus interactions despite being dispensable for replication in vitro [50,51]. In addition, positioning the reporter cassette upstream of the *N* gene may facilitate robust reporter expression because the *N* gene is transcribed from one of the most abundant PEDV subgenomic RNAs (sgRNAs).

Nevertheless, although the M-N region represents a promising insertion site for reporter expression, insertion of a foreign sequence into this region may still influence viral replication. Coronavirus gene expression relies on discontinuous transcription mediated by transcription regulatory sequences (TRSs), and the addition of the EGFP-2A cassette may alter local RNA secondary structures, TRS accessibility, or sgRNA synthesis. Furthermore, although the 2A peptide is expected to mediate efficient co-translational separation of EGFP and N protein through ribosomal skipping, incomplete cleavage cannot be ruled out and may influence N protein-related functions involved in viral RNA binding, replication complex formation, and virion assembly. In addition, the increased genome length associated with the reporter cassette may impose a modest fitness cost during viral RNA replication or genome packaging. These factors could therefore have contributed, at least in part, to the slight reduction in viral replication and pathogenicity observed for rPEDV-EGFP compared with the parental wild-type PEDV-BJ-2023 strain. However, the recombinant virus retained stable EGFP expression during serial passage, productive replication in vitro, and pathogenicity in vivo, supporting the feasibility of the M-N region as a potential site for PEDV reporter virus construction. Despite these encouraging results, it is important to acknowledge that reporter viruses may be subject to genetic instability or attenuation during more extensive serial passages. In addition, the relatively small sample size used in animal experiments (*n* = 3 per group) may have limited the statistical power and increased the impact of interindividual variation; therefore, the in vivo findings should be interpreted with appropriate caution. Despite these limitations, the reporter virus described here represents a valuable tool for applications including antiviral screening and real-time visualization of infection dynamics.

In summary, we report the rapid construction of a full-length infectious clone of a G2c PEDV strain using a TAR-based strategy and demonstrate its utility for virus rescue and genome manipulation. This system expands opportunities for studying the biology of emerging PEDV variants and supports future development of next-generation vaccines and antiviral interventions.

## 5. Conclusions

In conclusion, we successfully isolated an emerging G2c PEDV field strain (PEDV-BJ-2023) and established a full-length infectious cDNA clone using TAR cloning in yeast. The recombinant viruses displayed virion morphology and plaque characteristics similar to those of the parental wild-type PEDV-BJ-2023 strain, while the rPEDV-EGFP reporter virus maintained stable EGFP expression over eight serial passages. All rescued viruses remained pathogenic in piglets, causing severe diarrhea, weight loss, and characteristic intestinal lesions, consistent with the parental PEDV-BJ-2023 strain. However, the insertion of the EGFP-2A cassette may subtly affect viral replication and pathogenicity. This reverse genetics platform provides a valuable tool for investigating the biology of emerging G2c PEDV strains and supports the development of next-generation vaccines and antiviral strategies.

## Figures and Tables

**Table 1:** Primers used for amplification of PEDV genome fragments.

Primer	Sequence (5′→3′)
PEDV-F1	ACTTAAAAGATTTTCTATCTACG
PEDV-R1	AACAACATTAAAGACCTTAAGAC
PEDV-F2	CACGGTCCCATTAAAGTT
PEDV-R2	TGTATTTGCTATCTGTTATGTCA
PEDV-F3	TTGCAACTGAAGTTGGTA
PEDV-R3	ATAGTGTTACAACCACCAT
PEDV-F4	TCCATTAAGGCAGAAGGA
PEDV-R4	TGTGGCGGTAGTTTTATAT
PEDV-F5	TTGCTCAAATGGGAAGTC
PEDV-R5	GTAAATAGAAGGCATGGAATAA
PEDV-F6	TAAAATGTGGAGGTGGATG
PEDV-R6	AGATAAGTGGCATTAAAAAC
PEDV-F7	CAGATTACATCGATGTCAACAA
PEDV-R7	GTGTATCCATATCAACACC

**Table 2:** Primers used for assembly of the full-length PEDV infectious cDNA clone.

Primer	Sequence (5′→3′)
pYES1L-F	CAAAAAAAAAAAAAAAAAAAAAAAAAAAAAAAAAGGCCG
pYES1L-R	TAAGTCTATTAGAGCTCAAGCTCTG
pYES1L-F1	TCTATATAAGCAGAGCTTGAGCTCTAATAGACTTAAAAGATTTTCTATCTACGGAT
pYES1L-F2	GATGCTGCTATGGCTATTGAT
pYES1L-F3	CGTGGCGTTGCTGATAAGG
pYES1L-F4	GCAAGGTTTGTGGTTGTTGG
pYES1L-F5	CGTATGTTTTTGGCTAAAAATCCAAGATGGTCAAA
pYES1L-F6	GAATGGTAAGTTGCTAGTGCG
pYES1L-F7	AGGTTGTTGTAGGGGTCCT
pYES1L-R1	TGACGACCATAACCATCAATAGC
pYES1L-R2	TTAAAAATGCAAGCGCCCT
pYES1L-R3	GCAGCCATTAGACAGCCAAC
pYES1L-R4	TTTGACCATCTTGGATTTTTAGCCAAAAACATACG
pYES1L-R5	TGGCGTCATTATTACGCACTAG
pYES1L-R6	CGTAAGGTTGAAGTCTAGGACC
pYES1L-R7	CCTTTTTTTTTTTTTTTTTTTTTTTTTTTTTTTTTGTGTATCCATATC
cPCR-F1	GTCTGTGGTTGTGGTACTG
cPCR-F2	GCTACATAGGTGCCACTG
cPCR-R1	GTCAATGGCCTTTGCTATG
cPCR-R2	GCCTAGACAAGCAACATC

**Table 3:** Scoring criteria for clinical symptoms in PEDV-infected piglets.

Clinical Signs	Criteria	Score
Diarrhea	Normal	0
	Soft feces	1
	Soft feces with watery content	3
	Watery diarrhea	4
Activity level	Normal	0
	Slow movement, lethargic	1
	Lying down, depressed	3
	Prostrate, moribund	4
Vomiting	None	0
	Occasional vomiting	1
	Vomiting several times per day	3
	Frequent, uncontrollable vomiting	4

## Data Availability

Data will be provided on request.
